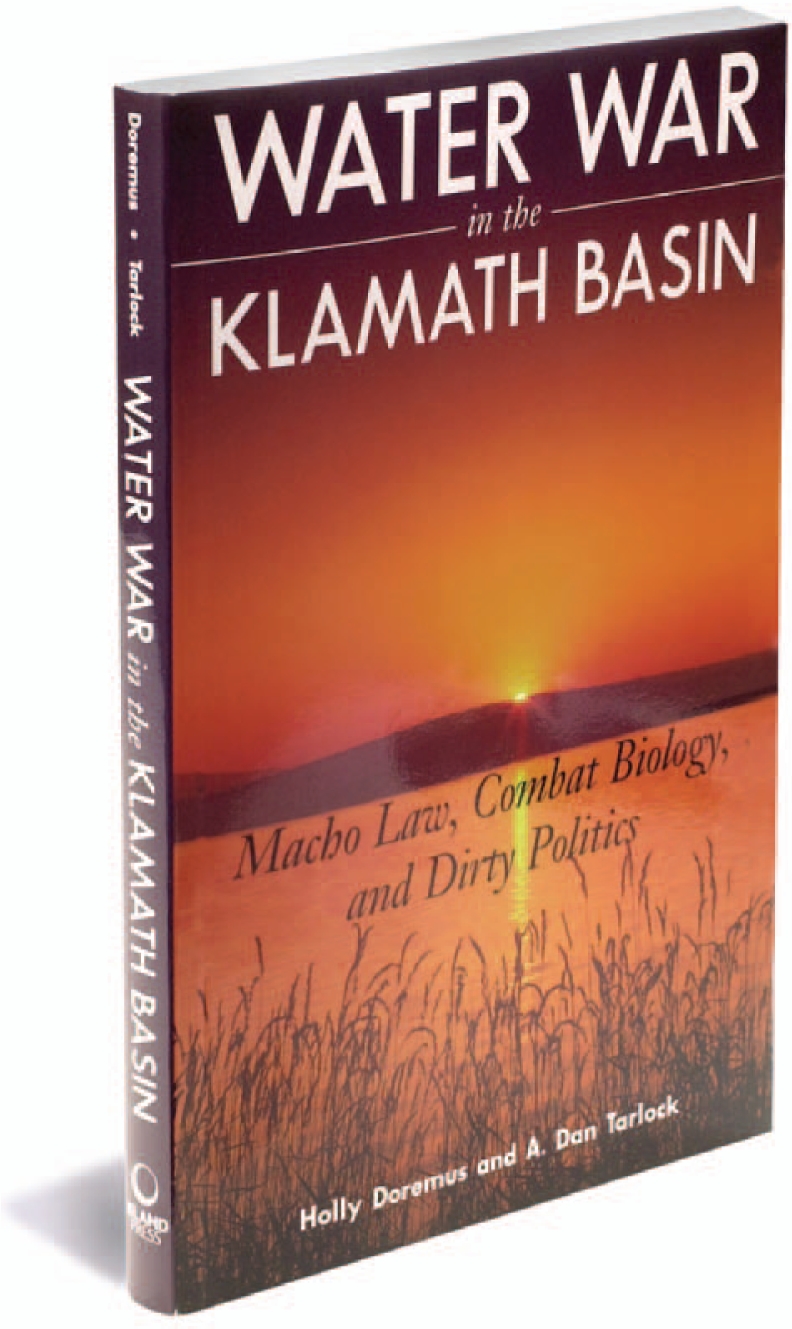# Water War in the Klamath Basin: Macho Law, Combat Biology, and Dirty Politics

**Published:** 2008-09

**Authors:** John Krist

**Affiliations:** During a journalism career that spanned nearly 25 years, John Krist won numerous awards for his reporting and commentary on land-use policy, natural resources, and environmental issues. His 2004 series on water in the West, which included coverage of the Klamath Basin conflict, was honored as the year’s best agricultural and natural resources reporting by the California Newspaper Publishers Association

In July 2001, employees of the U.S. Bureau of Reclamation closed the big steel gates across the intake of a major irrigation canal just outside the town of Klamath Falls, Oregon. Water stopped flowing to about 85% of the farms in the Klamath Basin, a high-elevation bowl ringed by forested mountains on the Oregon–California border. The ostensible purpose of the shutdown was to protect three threatened or endangered fish species living in the lakes and rivers tapped by the irrigation system.

That water cutoff, coming in the middle of the irrigation season in a region almost wholly dependent on irrigated agriculture, ignited a legal and political firestorm. Hundreds of outraged farmers and their supporters surrounded the main irrigation canal’s control structure and turned the water back on, retreating only when federal law enforcement agents arrived. Lawsuits were filed. Protesters arrived from as far away as Nevada, Montana, and Malibu. Among their props was a giant metal bucket, a symbolic water delivery to parched Klamath farms. Three years later, although the out-of-town horde drawn by the water shutoff had long since departed, the bucket—with an American flag on top and the words “Klamath Bucket Brigade” painted on the side—still greeted visitors in front of the County Government Center in Klamath Falls.

The dramatic standoff and its bitter aftermath drew nationwide media attention. Nearly every story printed or broadcast during that long, drought-parched summer focused on the spectacle of “farms versus fish,” an emotionally resonant but not particularly accurate or useful description of the conflict.

*Water War in the Klamath Basin: Macho Law, Combat Biology, and Dirty Politics,* a new book by a pair of veteran environmental-law experts, delves deep into the tangled legal underpinnings of the conflict, revealing it to have been about much more than farms and fish. Holly Doremus, a law professor at the University of California at Davis, and A. Dan Tarlock, distinguished professor of law at the Chicago-Kent College of Law, have crafted a dense but concise text that describes the assumptions underlying federal and state natural-resource policies and explains how legal contradictions, changing cultural values, and evolving scientific understanding combined to make the clash in the Klamath Basin both inevitable and intractable.

The Klamath Basin contains about 1,400 farms encompassing more than 200,000 acres of cropland, most of it served by a large and complex federal irrigation system known as the Klamath Project. It is one of the oldest federal water projects in the nation, and like nearly all the great public works of the Reclamation Era, it was constructed with no concern for the effect its dramatic reworking of natural hydrology would have on the fish and other wildlife occupying the region’s lakes, streams, and wetlands.

The 2001 water cutoff, at least superficially, represented a belated effort to mitigate the effect of agricultural irrigation diversions on three fish species: the Lost River and shortnose suckers, inhabitants of the basin’s largest lake and its tributaries; and coho salmon, found in the Klamath River downstream from the project. But as Doremus and Tarlock correctly point out, the Klamath conflict also pitted the interests of farmers and ranchers against those of commercial fishermen on the coast more than 200 miles away. It was about the conflicting cultural values of whites and Native Americans. And finally, it was about two contradictory ways of thinking about the environment: one deriving from the past and viewing natural resources from a purely utilitarian perspective, and one that arose only recently in American history, regarding ecosystems and their living components as having rights and value beyond their economic utility to human beings.

*Water War in the Klamath Basin* is sometimes a tedious read—the authors have an annoying habit, useful in introductory college textbooks but nowhere else, of reminding the reader about what they have already explained and previewing matters they will explain later—and it shortchanges the rich human component of the Klamath saga. But it is a useful and thorough primer on western water law and federal environmental policy. And it serves as a cautionary guide to the conflicts that inevitably will arise in other overallocated watersheds as population growth, a warming climate, and failing ecosystems conspire to reveal profound flaws in the hydraulic foundation of the West.

## Figures and Tables

**Figure:**